# Four year surveillance of central line-associated bloodstream infection (CLABSI) in neonatal intensive care unit (NICU)

**DOI:** 10.1186/1753-6561-5-S6-O8

**Published:** 2011-06-29

**Authors:** A Décary, R Mandel, R Rodrigues, C Frenette, B Lussier

**Affiliations:** 1 Infection Control, McGill University Health Center, Montreal, Canada

## Introduction / objectives

From 2007-11 surveillance for CLABSI was performed in our tertiary level, 26 bed NICU. As CLABSI is a preventable nosocomial infection, we are reporting the impact of introducing a Peripherally Inserted Central Catheter (PICC) interprofessional team, dedicated to implement a prevention bundle for CLABSI since 2007.

## Methods

All laboratory confirmed blood cultures from the NICU were evaluated to determine CLABSI using the National Healthcare Safety Network (NHSN) definition prior and after 2008.The PICC team are specific bedside nurses trained to insert, monitor insertion site and line removal. They use age/weight specific skin antiseptics (2% CHG, 0.5% and 2% CHG in 70% alcohol) with designated equipment. Bedside nurses access infusion lines for administering medication, total parenteral nutrition and changing the line/connectors.

## Results

Over the past 4 years (2007–11) there were 37 CLABSI using the old NHSN definition versus 22 CLABSI with the new definition. The infection rate was 5.97 /1000 Catheter Days (CD) and 3.55/1000 CD respectively. The overall catheter utilization ratio was 0.20.The most prevalent microorganisms recovered were *Coagulase-negative Staphylococci* (71%) followed by *Candida*, *Enterobacter* and *Klebsiellaspecies* (6% each).There were two associated CLABSI mortalities within 30 days. Rates of CLABSI decreased from 8.1 to 3.71 using old definition and from 4.7 to 1.06 per 1000 CD using new definition 2007-08 to 2010-11.Catheter utilization ratio increased from 0.19 to 0.24.

## Conclusion

Implementation of a dedicated interprofessional PICC Team and a prevention bundle was successful in decreasing rates of CLABSI in the NICU. The change in definition considerably affects the rates of CLABSI (-41%).

## Disclosure of interest

None declared.

